# Switching Hedgehog inhibitors and other strategies to address resistance when treating advanced basal cell carcinoma

**DOI:** 10.18632/oncotarget.28080

**Published:** 2021-09-28

**Authors:** Hung Q. Doan, Leon Chen, Zeena Nawas, Heng-Huan Lee, Sirunya Silapunt, Michael Migden

**Affiliations:** ^1^Department of Dermatology, Division of Internal Medicine, The University of Texas MD Anderson Cancer Center, Houston, TX, USA; ^2^Department of Dermatology, University of Texas McGovern Medical School, Houston, TX, USA; ^3^US Dermatology Partners, Houston, TX, USA; ^4^Department of Dermatology, Baylor College of Medicine, Houston, TX, USA; ^5^Department of Molecular and Cellular Oncology, The University of Texas MD Anderson Cancer Center, Houston, TX, USA; ^6^Departments of Dermatology and Head and Neck Surgery, The University of Texas MD Anderson Cancer Center, Houston, TX, USA

**Keywords:** basal cell carcinoma, Hedgehog pathway inhibitor, resistance, switching, combination therapy

## Abstract

Although basal cell carcinoma (BCC) is often managed successfully with surgery, patients with locally advanced BCC (laBCC) or metastatic BCC (mBCC) who are not candidates for surgery or radiotherapy have limited treatment options. Most BCCs result from aberrant Hedgehog pathway activation in keratinocyte tumor cells, caused by sporadic or inherited mutations. Mutations in the patched homologue 1 gene that remove its inhibitory regulation of Smoothened homologue (SMO) or mutations in *SMO* that make it constitutively active, lead to Hedgehog pathway dysregulation and downstream activation of GLI1/2 transcription factors, promoting cell differentiation and proliferation. Hedgehog inhibitors (HHIs) block overactive signaling of this pathway by inhibiting SMO and are currently the only approved treatments for advanced BCC. Two small-molecule SMO inhibitors, vismodegib and sonidegib, have shown efficacy and safety in clinical trials of advanced BCC patients. Although these agents are effective and tolerable for many patients, HHI resistance occurs in some patients. Mechanisms of resistance include mutations in SMO, noncanonical cell identity switching leading to tumor cell resistance, and non-canonical pathway crosstalk causing Hedgehog pathway activation. Approaches to managing HHI resistance include switching HHIs, HHI and radiotherapy combination therapy, photodynamic therapy, and targeting Hedgehog pathway downstream effectors. Increasing understanding of the control of downstream effectors has identified new therapy targets and potential agents for evaluation in BCC. Identification of biomarkers of resistance or response is needed to optimize HHI use in patients with advanced BCC. This review examines HHI resistance, its underlying mechanisms, and methods of management for patients with advanced BCC.

## INTRODUCTION

Basal cell carcinoma (BCC) is the most common keratinocyte tumor and human malignancy worldwide [1, 2]. In the United States, the overall lifetime risk of developing BCC is estimated to be at least 20%; the incidence rate and the associated healthcare costs increase annually [3]. Major risk factors for the development of BCC include age and ultraviolet light exposure [3]. Most cases of BCC are treated surgically, which typically has an excellent prognosis [4, 5]. Standard excision or curettage and electrodessication are often used for low-risk lesions, and Mohs micrographic surgery is often effective for treating high-risk tumors [4]. However, a subset of advanced BCC cases that includes both locally advanced BCC (laBCC) and metastatic BCC (mBCC) are not amenable to surgery due to high morbidity and risk for severe disfigurement [1, 6].

**Figure 1:**
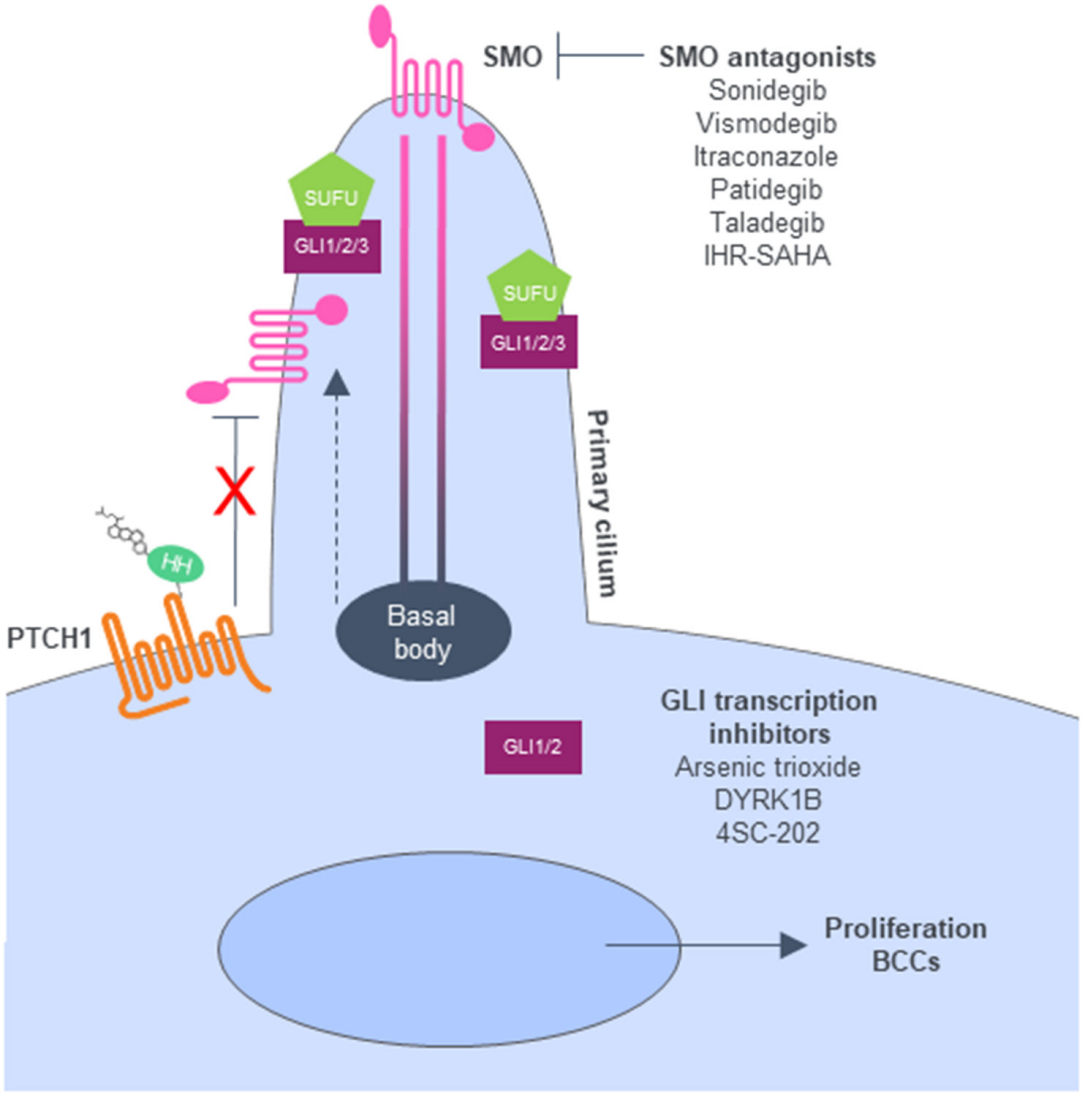
Hedgehog signaling pathway. When the HH ligand is present, Ptch1 is inactivated and allows SMO to move to the top of the cilia, triggering downstream signals and GLI activation. Adapted from [82]. Abbreviations: BCC: basal cell carcinoma; DYRK1B: dual-specificity-tyrosine-phosphorylation-regulated kinase 1B; GLI: glioma-associated oncogene; HH: Hedgehog; Ptch1: Patched-1; SMO: Smoothened; SUFU: Suppressor of fused.

The majority of BCCs have aberrant activation of the Hedgehog signaling pathway, most often from inactivating mutations in a negative regulator, patched homologue 1 (*PTCH1*), or less often from activating mutations in a positive regulator, Smoothened homologue (*SMO*) [7, 8]. Dysregulation of the Hedgehog signaling pathway leads to activation of transcription by the transcription factors GLI1 and GLI2 (Figure 1) [9]. In keratinocytes, the Hedgehog signaling pathway controls cell differentiation and proliferation to maintain cutaneous stem cell populations and regulate the development of sebaceous glands and hair follicles [10].

Hedgehog pathway inhibitors (HHIs) are the only US Food and Drug Administration (FDA)-approved pharmacologic treatment for patients with advanced BCC [4]. Vismodegib (Erivedge^®^, Genentech Inc., South San Francisco, CA) and sonidegib (ODOMZO^®^, Sun Pharmaceutical Industries, Inc., Cranbury, NJ, USA) are two oral HHIs indicated for the treatment of adults with laBCC following surgery or radiation therapy or for those who are not candidates for surgery or radiation therapy [11, 12]. Vismodegib is also indicated for the treatment of adults with mBCC in the US [12]. These agents are small molecule inhibitors of SMO that prevent the activation of GLI1 and GLI2.

The efficacy and safety of vismodegib was investigated in three main clinical trials: the ERIVANCE study, the STEVIE study, and the MIKIE study [13–15]. Sonidegib was evaluated in the phase 2 BOLT clinical trial at 6, 12, 30, and 42 months [16–19].

The phase 2, multicenter, international, non-randomized ERIVANCE study (NCT00833417) evaluated a continuous regimen of vismodegib 150 mg/day in 63 patients with laBCC and 33 patients with mBCC [13]. In the primary analysis, objective response rate (ORR) by independent review, was 43% for patients with laBCC and 30% for patients with mBCC; in both cohorts, most patients had tumor shrinkage. The most common grade 3–4 adverse events (AEs) were muscle spasms, weight loss, fatigue, and loss of appetite; the rate of discontinuation due to AEs was 12% [13].

The STEVIE study (NCT01367665), a phase 2, single-arm, multicenter, international, non-randomized, open-label study, evaluated vismodegib in elderly patients (median age of 72 years) with a high incidence of comorbidities—a population similar to clinical practice. Patients with laBCC (*n* = 1119) or mBCC (*n* = 96) received vismodegib 150 mg/day [14]. The primary endpoint was safety. The most common treatment-emergent AEs (TEAEs; ≥ 20% of patients) were muscle spasms, alopecia, dysgeusia, and decreased weight, similar to the profile observed in ERVIANCE [13, 14]. Vismodegib exposure ≥ 12 months did not result in increased incidence or severity of TEAEs, and the rate of discontinuation due to TEAEs was 31%. Among patients with measurable disease at baseline, investigator-assessed responses occurred in 68.5% of patients with laBCC and 36.9% of patients with mBCC [14].

The randomized, double-blind, regimen-controlled, intermittent dosing, phase 2 MIKIE study (NCT01815840), investigated vismodegib in adult patients with multiple BCCs amenable to surgery, as well as patients with basal cell nevus syndrome [15]. Patients were randomized 1:1 to treatment A (150 mg oral vismodegib once daily for 12 weeks, followed by three rounds of 8 weeks of placebo daily, then 12 weeks of 150 mg vismodegib once daily [*n* = 116]) or treatment B (150 mg oral vismodegib once daily for 24 weeks, then three rounds of 8 weeks of placebo daily followed by 8 weeks of 150 mg vismodegib once daily [*n* = 113]) [15]. The primary endpoint was percent reduction from baseline in clinical BCCs at week 73 [15]. Mean percent reduction in BCCs from baseline to the end of the study was 62.7% (95% confidence interval [CI] 53.0–72.3) and 54.0% (43.6–64.4) for treatment groups A and B, respectively.[15] Overall, 94% and 97% of patients in treatment groups A and B, respectively, experienced TEAEs, while the most common AEs grade 3 or greater included muscle spasms, increased blood creatine phosphokinase, and hypophosphatemia [15].

The safety and efficacy of sonidegib, another FDA-approved HHI for advanced BCC, was evaluated in the phase 2, multicenter, randomized, double-blind BOLT clinical study (NCT01327053) [11, 16–19]. Patients with laBCC not amenable to surgery or radiotherapy or mBCC received sonidegib 200 mg (*n* = 79) or 800 mg (*n* = 151) once daily. In the primary analysis, the primary endpoint, ORR by central review, was 43% vs 15% for laBCC and mBCC, respectively, in the 200-mg group, and 38% vs 17%, respectively, in the 800-mg group [19]. The most common AEs included muscle spasms, dysgeusia, alopecia, and nausea. The most common AEs leading to treatment discontinuation were muscle spasm, dysgeusia, weight decrease, and nausea [16–19].

Other HHIs under investigation in advanced BCC include patidegib, itraconazole, and arsenic trioxide. Patidegib has orphan drug and breakthrough therapy designations from the FDA and an orphan drug designation from the European Medicines Agency as a topical agent for Gorlin syndrome, also known as nevoid basal cell carcinoma syndrome, a rare genetic form of BCC characterized by mutations in *PTCH1* resulting in multiple BCCs [20, 21]. Patidegib is being studied in a phase 3 trial for the reduction of disease burden in Gorlin syndrome (NCT03703310) and in a phase 2 trial of non-Gorlin high frequency BCC (NCT04155190). Itraconazole binds to SMO at a site distinct from the other HHIs [22]. Arsenic trioxide destabilizes GLI2 to inhibit transcription of target genes of the Hedgehog signaling pathway and has shown activity in combination with itraconazole in BCC refractory to vismodegib or both vismodegib and sonidegib [23].

Despite the reported excellent overall efficacy of and safety profile for treatment with vismodegib and sonidegib, some patients demonstrate tumor intrinsic resistance to HHI treatment or develop resistance during treatment. This review will discuss HHI resistance, its underlying mechanisms, and methods of management for patients with advanced BCC.

### Hedgehog inhibitor resistance in clinical studies and practice

Results from clinical studies of vismodegib and sonidegib show proportions of patients with advanced BCC resistant to treatment (intrinsic resistance) and who develop resistance to treatment after an initial response (acquired resistance). Case studies from clinical practice provide further descriptions on lack of response, partial response, and development of resistance after an initial response to an HHI [24, 25].

In the primary analysis of the ERIVANCE study, the rate of progressive disease (PD) with vismodegib treatment was 13% and 3% for laBCC and mBCC, respectively [13]. In both cohorts, median progression-free survival (PFS) was 9.5 months, and median duration of response was 7.6 months [13]. At data cutoff (9 months after last patient enrolled), 10 of 13 patients who had a complete response did not have disease progression. Disease progression was the most common reason for discontinuation of vismodegib among patients with mBCC (18%); 7% of patients with laBCC also discontinued treatment for that reason [13].

In the primary analysis of the BOLT study, the rate of PD for patients receiving sonidegib 200 mg was 10% for laBCC and 8% for mBCC [19]. Median PFS was not reached in patients with laBCC and was 13.1 months in patients with mBCC in the 200-mg group; it was not reached in patients with laBCC and was 7.6 months in patients with mBCC in the 800-mg group. Median duration of tumor response was not reached for either disease cohort with sonidegib 200 mg or for patients with laBCC in the 800-mg group, and was 8.3 months for patients with mBCC in the 800-mg group [19]. At the 12-month analysis, 29.1% of patients receiving sonidegib 200 mg and 9.9% of patients receiving sonidegib 800 mg discontinued treatment due to PD [17].

A case report described an elderly man with recurrent laBCC invading his right orbit and frontal sinus after excision and radiation therapy [25]. After 1 year of treatment with vismodegib 150 mg/day, the lesion initially decreased in the nasal region, but later progressed in the orbital region and upper cheek [25]. After receiving treatment with vismodegib for a total of 28 months, molecular analysis of tumor tissue revealed a mutation in *PTCH1* but no mutation in *SMO* [25]. After discontinuing vismodegib treatment, he developed mBCC. Thus, BCCs may exhibit intratumoral heterogeneity and may acquire mutations during treatment that confer HHI resistance [25].

Some patients develop resistance after an initial response to an HHI [26–28]. In the STEVIE study, a 59-year-old man with Gorlin syndrome responded to vismodegib 150 mg/day and achieved a reduction in size and in number of BCCs, until clinically undetectable, and a disappearance of palmar pits. After 3 years of continuous vismodegib treatment, three lesions appeared (2 histologically confirmed as BCC recurrence), and treatment was discontinued per study protocol. Within 2 months, multiple recurrences were observed at the original locations. Molecular analysis of the two excised BCCs showed a germline mutation in *PTCH1* and a mutation in *SMO* known to cause vismodegib resistance [26].

Although biomarkers of HHI resistance have not been identified, hair regrowth may be an early marker of HHI resistance based on observations in a 65-year-old female with Gorlin syndrome and a 56-year-old male with laBCC. Each had a partial response to vismodegib and alopecia as a treatment side effect. After being progression-free on long-term continuous vismodegib treatment, each patient experienced disease progression. In addition, each patient had reversal of alopecia within 2 months of disease progression, suggesting resistance to vismodegib allowed for sufficient Hedgehog pathway signaling to reverse the alopecia and enable tumor growth [29].

### Mechanisms of Hedgehog inhibitor resistance

HHI resistance may develop via disruption of the HHI binding site through *SMO* mutations, which differentially modulate the activity of HHIs depending on changes in SMO structure [30–32]. *In vitro* binding site kinetics of vismodegib and other SMO antagonists (with the same binding site) differed between wild type *SMO* and a frequently identified SMO mutant (D473H) known to cause vismodegib resistance; moreover, these binding differences may provide an explanation for sensitivity to D473H and SMO D473H inhibitory activity [31].

Most patients with HHI-resistant BCC have mutations in *SMO*, regardless of whether resistance is intrinsic or acquired [33–35]. Molecular analysis of tumors from two vismodegib-resistant patients enrolled in the STEVIE study—one with intrinsic resistance and one with acquired resistance—showed the patient with intrinsic resistance had an SMO G497W mutation known to interfere with drug entry to the binding site, and the patient with acquired resistance had an SMO D473Y mutation known to interfere directly with vismodegib binding affinity [35]. Analysis of *SMO* mutations identified in 50% of SMO inhibitor-resistant BCCs demonstrated two distinct mechanisms of resistance: binding site mutations, observed in patients with acquired resistance, or mutations releasing SMO autoinhibition to confer constitutive SMO activity, observed in patients with intrinsic and acquired resistance [36]. Concurrent, non-SMO mutant forms of resistance were identified as reduced copy numbers of Suppressor of fused (SUFU) and increased copy numbers of GLI2 [34].

Typically, canonical activation of the Hedgehog signaling pathway occurs in cancers with mutations in *PTCH1* and *SMO*, which are often responsive to SMO antagonists [37]. However, HHI resistance may occur with noncanonical Hedgehog pathway activation. Specifically, regulation and activity of GLI expression can occur in response to pathways other than PTCH and SMO, thereby reducing the efficacy of SMO antagonists [37]. Recently, a link between cytoskeletal regulators and Hedgehog pathway activation was identified in SMO inhibitor-resistant BCC [38]. In these SMO inhibitor-resistant tumors, Rho-mediated activation of cytoskeletal F-actin to G-actin increased the Hedgehog signaling pathway activity through activation of the transcription factor serum response factor (SRF) and its coactivator megakaryoblastic leukemia-1 (MKL1) [38]. This increase in cellular SRF/MKL1 increased nuclear accumulation of SRF/MKL1 and potentiated GLI-mediated Hedgehog pathway activation. This SRF-MKL1 cytoskeletal signaling axis represents a therapeutic target for HHI-resistant BCCs. Most SMO inhibitor-resistant BCCs analyzed contained activated MKL1 in the nucleus, and MKL1 inhibitors had antitumor activity in explants of resistant BCCs [38].

Another mechanism of HHI resistance involves BCC switching between canonical and noncanonical Hedgehog pathways [33, 39–41]. One study found that residual BCCs initiated a different transcriptional program compared with untreated BCCs, and this identified switch was linked to Wnt pathway activation. When vismodegib was combined with a Wnt pathway inhibitor, BCC tumor burden was reduced [40]. The Wnt pathway was observed as a mechanism for tumor cells to evade vismodegib and maintain a quiescent state independent of Hedgehog pathway signaling [40]. Further investigation of Wnt pathway-mediated evasion demonstrated that BCCs induced in a genetic mouse model of BCC expressed the leucine-rich repeat-containing G-protein coupled receptor Lgr5; upon drug withdrawal, the proliferation of these persistent, Lgr5-expressing cells promoted tumor regrowth [41]. Therefore, the Wnt-activated and Lgr5-positive cells represent a type of resistance that may be considered tumor persistent rather than tumor progressive while patients are being treated with HHIs.

### Switching Hedgehog inhibitors in clinical studies and practice

One approach to addressing HHI resistance in patients with BCC is switching agents after intrinsic or acquired resistance becomes apparent. Clinical study results and findings of case reports on switching HHIs are summarized in Table 1.

**Table 1:** Summary of clinical studies and case reports on switching Hedgehog inhibitors

Publication	Type	Patient characteristics	Treatment	Efficacy results	Safety results
Yoon et al. [42]	Case report	• 87-year-old male • Inoperable laBCC of ethmoid sinus and brain • Treated with vismodegib 150 mg/day 5 years earlier; acquired resistance after > 1 year, vismodegib was discontinued • Radiation therapy • Recurrence after 2 years • Progression on 6 months of vismodegib • Pembrolizumab (3 cycles) resulted in further progression	Sonidegib 200 mg/day + itraconazole pulse dosed at 100 mg/day for 2 weeks/rest for 2 weeks, repeated monthly	• Significant improvement after 3 months • After 8 months, intracranial lesion no longer visible, intranasal and sinus lesions stable/slightly improved	No major AEs
Tran et al. [43]	Clinical study	• laBCC or mBCC • Median (range) age at enrollment, 61 (45–87) years • 5 patients had received prior HHIs (vismodegib, taladegib)	Sonidegib 200 mg/day + buparlisib 80 mg/day, 28-day cycles	• Median (range) follow-up, 8 (0.5–20) months • 7 evaluable patients • ORR: 1/7 (14.3%) • PR/SD as best response: 5/7 (71%)	Grade 3 TRAEs 50% patients led to early study termination
Zargari et al. Dermatol Ther. 2017	Case report	• 48-year-old male • Infiltrative BCC on the nose excised incompletely • Recurrence and appearance of new lesions treated with radiation therapy • Recurrent lesion improved with imiquimod treatment • Recurrent lesion infiltrated the whole nose, visual limitation • Treated with itraconazole twice daily for 4 months with minimal effect	Vismodegib 150 mg/day for 3 consecutive 28-day cycles	• Significant decrease in size and signs of healing at 1 month, improved visual movement • No signs of recurrence 8 months after treatment	No significant AEs
Danial et al.[44]	Clinical study	• Advanced BCC with demonstrated intrinsic or acquired resistance to vismodegib • Mean (range) age, 57.4 (42–91) years	Sonidegib 800 mg/day, 28-day cycles	• Median (range) treatment duration, 6 (3–58) weeks • 5/9 patients had PD within a median of 6 weeks • 3/9 patients had SD within a median of 4 weeks • 1 patient discontinued treatment for grade 3 rhabdomyolysis	Grade 3 AEs: rhabdomyolysis, nausea, altered mental status
Zhu et al.[48]	Case report	• Male in his 50s • Gorlin syndrome • Multiple BCCs on the head, neck, trunk, and all 4 extremities • Cutaneous lesions shrank with saridegib 130 mg/day • Lung metastases were refractory to saridegib 130 mg/day	Vismodegib 150 mg/day	• Vismodegib 150 mg/day resulted in further shrinkage of cutaneous lesions • After 4 months of treatment, lung metastases had progressed	Not reported

Abbreviations: AE: adverse event; BCC: basal cell carcinoma; laBCC: locally advanced BCC; mBCC: metastatic BCC; ORR: overall response rate; PD: progressive disease; PR: partial response; SD: stable disease; TRAE: treatment-related adverse event.

A patient with laBCC and acquired resistance to vismodegib was ultimately administered combination therapy with two other SMO inhibitors that bind to SMO on different sites than vismodegib and from each other. In this patient, daily dosing of sonidegib 200 mg with concomitant pulse dosing of itraconazole 100 mg/day caused substantial regression of BCC lesions after 3 months, with a noted further improvement after 8 months, and the treatment was well tolerated. This shows that a positive response with other SMO inhibitors after one has failed is possible [42].

Because phosphoinositide-3-kinase (PI3K) is implicated in SMO inhibitor resistance, the efficacy and safety of combination therapy with 28-day cycles of sonidegib 200 mg/day and a pan-PI3K inhibitor, buparlisib, at a dose of 80 mg/day, were investigated in an open-label, single-arm clinical trial. After a total of eight grade 3 AEs in 50% of the safety population, the study was terminated early. Overall, seven patients were evaluated for efficacy, five with previous HHI failure. This combination achieved an overall response rate of 14.3% and a disease control rate of 71%; one patient had a partial response, and four patients remained with stable disease [43].

In contrast, nine patients with intrinsic or acquired resistance to vismodegib treatment received sonidegib 800 mg/day. Overall, three patients had stable disease, one response was unknown, and five had PD, suggesting resistance to vismodegib conferred resistance to sonidegib [44]. This study was limited due to a relatively short treatment duration; of the nine patients in this trial, only one patient was treated beyond 14 weeks (total of 58 weeks), and some patients were treated for only 3 weeks. Therefore, this treatment period may not have been sufficient to achieve a measurable clinical response to sonidegib. In addition, this patient who had a D473H mutation in SMO was treated for 58 weeks and experienced disease progression. It has been shown *in vitro* that the affinity (pKi) of vismodegib and sonidegib for SMO decreased significantly compared with wild-type, from 8.32 to 5.95 (>100-fold) and from 7.68 to 6.91, respectively, in the presence of a D473A mutation [45]. In contrast, in the presence of the E518A mutation, the pKi for vismodegib decreased to 6.68 and increased somewhat for sonidegib. Consequently, if resistance to vismodegib is due to a E518A mutation, treatment with sonidegib may still be effective. The affinity of the SMO antagonist LY2940680, taladegib, was not affected by the D473H mutation, even though taladegib and vismodegib have 14 contact residues in common [45]. This supports the response to taladegib with a mutation rendering resistance to vismodegib [46]. Based on the SMO crystal structure, the computational docking of vismodegib onto SMO demonstrated that the locations of the SMO-W281, SMO-V321, SMO-I408 and SMO-C469 mutations are near the drug-binding pocket [34, 47]. These mutations disrupt the hydrophobic pocket, interfere with the positioning of adjacent binding residues, change the conformation of the residues, and have steric effects on the binding pocket, respectively, to affect vismodegib binding [34]. Whether a patient who develops resistance to a specific HHI due to an SMO mutation will respond to a different HHI depends on the specific mutation, binding location, and whether a conformational change is generated to prevent drug binding in a direct or indirect manner. Along with patient sequencing data, these findings may help predict patient response to a subsequent HHI after failure of initial HHI treatment.

Vismodegib treatment in a patient diagnosed with Gorlin syndrome resulted in regression of cutaneous BCCs but disease progression of mBCCs in the lung, a response similar to that experienced with prior saridegib treatment. Consequently, since both HHIs produced a differential clinical response, with some tumors responding and others (metastatic) with no response, BCCs in patients with Gorlin syndrome may not be genetically identical [48]. Moreover, this patient’s previous exposure of chemotherapy to treat testicular cancer may have conferred resistance to SMO inhibitors [48].

### Additional strategies for managing Hedgehog inhibitor resistance

Combination therapy is a strategy to reduce the potential of acquired resistance. Use of combination therapy targeting different mechanisms of action may reduce the chance of acquired resistance while increasing efficacy. Several case reports and small analyses discuss the potential benefits of an HHI in combination with radiotherapy, photodynamic therapy (PDT), or other HHIs [23, 49–55]. Administration of vismodegib 150 mg/day in combination with radiotherapy resulted in a complete response and near-complete skin closure of multiple massive BCCs on the torso of a man in his 50s at 6 months following treatment [49]. In two patients who had recurrent advanced BCCs on the face, the combination of vismodegib 150 mg/day and radiotherapy showed no evidence of PD at last follow-up (9–12 months) [54]. In a retrospective analysis with a median follow-up of 12.5 months, four patients with inoperable laBCC received vismodegib 150 mg/day along with radiotherapy. Of the four patients, three had a persistent complete response, one was progression-free for 6 months, and all tolerated therapy well [53]. In an open-label pilot study of four male patients with multiple BCCs, patients were treated with combination therapy consisting of 3 continuous months of vismodegib 150 mg/day in combination with three consecutive sessions of PDT with topical application of 20% 5-aminolevulinic acid; results demonstrated a 90% complete response rate, a 10% partial response rate, and the treatment was well tolerated [50]. Combination HHI therapy with vismodegib 150 mg/day and itraconazole 100 mg/day for 4 months in a 71-year-old man with an invasive BCC led to a complete response and no clinical recurrence after treatment cessation at last follow-up of 18 months [51]. Five men with mBCC refractory to vismodegib or both vismodegib and sonidegib received arsenic trioxide 0.3 mg/kg daily for 5 days of a 28-day cycle and itraconazole 400 mg/day on non-arsenic trioxide days. Three evaluable patients had stable disease after three cycles of treatment and one patient had PD [23].

Another strategy to reduce the likelihood of developing acquired resistance is upfront shorter duration treatment prior to surgery, which may help reduce the time for acquired resistance to develop. HHIs are being studied as neoadjuvant therapy to reduce lesion size prior to surgery to optimize outcomes, as well as to enable the possibility of surgical excision in candidates previously unsuitable for surgery [56–66]. With this strategy, the development of HHI resistance may be less likely, as patients who respond to initial treatment will be able to have their tumors removed. In an open-label clinical study evaluating the use of vismodegib as neoadjuvant to surgery in high-risk patients with BCC, the study authors noted a mean decrease from baseline in target tumor surgical defect area of 27% in patients treated with at least 3 months of vismodegib at 150 mg/day [66]. Among eight patients in the trial who participated in a 2-year follow-up, neoadjuvant vismodegib reduced the surgical defect area from baseline by 34.8% and allowed for surgical clearance of the tumor with no recurrence at a mean follow-up of 22 months [62]. A retrospective chart review of patients who received HHI therapy for extensive BCC found reductions in tumor burden allowing for less extensive surgery than originally planned [65]. In addition, case studies report reductions in the size of BCCs prior to surgery with vismodegib therapy [58, 60, 64]: neoadjuvant vismodegib therapy decreased tumor size by at least 70% in patients with extensive BCCs [61, 63]. For patients with periocular tumors, neoadjuvant vismodegib treatment has enabled less radical, eye-sparing surgery [57, 59].

Targeting downstream effectors of the Hedgehog pathway is being studied in a clinical trial as a potential means to overcome or to prevent HHI resistance, as this approach directly interferes with multiple resistance pathways [67–72]. The GLI transcription factors are a logical target, as they are thought to regulate multiple pro-tumorigenic signaling pathways [67]. Another, more recently identified, target is the dual-specificity-tyrosine-phosphorylation-regulated kinase 1B (DYRK1B), which positively regulates GLI activity and can be inhibited by a small molecule inhibitor, DYRKi, in both SMO inhibitor-sensitive and -resistant cells [70]. Another small molecule inhibitor, 4SC-202, blocks Hedgehog/GLI signaling by targeting class 1 histone deacetylases (HDAC) in SMO inhibitor-sensitive and -resistant cells and is being studied clinically [69]. A new small molecule combinatorial SMO-HDAC antagonist that simultaneously inhibits SMO and GLI activity, IHR-SAHA, shows activity in SMO inhibitor-sensitive and -resistant cells. Epigenetic targeting is also being investigated, as bromo and extra C-terminal (BET) bromodomain proteins that regulate GLI transcription are inhibited by JQ1, a small molecule inhibitor of BRD4, in both SMO inhibitor-sensitive and -resistant cells [72].

Findings of a retrospective chart review and several case studies suggest targeting of programmed cell death protein 1 (PD-1) may be beneficial in patients with advanced BCC [73–78]. The chart review compared the incidence of BCC and squamous cell carcinoma among patients diagnosed with metastatic melanoma and treated with anti-PD-1 therapies, patients treated with other melanoma therapies, and patients with similar risk factors as a control group. The incidence of BCC was significantly lower among patients with melanoma receiving anti-PD-1 therapies vs controls, suggesting anti-PD-1 therapies may suppress BCC [74]. Characterization of PD-1 and PD-L1 expression patterns among BCC samples show PD-L1 expression on 22% of tumor cells and 82% of tumor infiltrating lymphocytes and macrophages, suggesting that treatment with immune checkpoint inhibitors may be effective in BCC [77]. Notably, several case reports of patients with laBCC or mBCC and HHI resistance have shown dramatic responses to treatment with either pembrolizumab or nivolumab [73, 76–78]. Although in one case, the nivolumab response was accompanied by the appearance of new superficial BCCs that could be treated with excision [76]. As of June 2021, three ongoing clinical trials of PD-1 inhibitors in patients with advanced BCC were registered with https://www.clinicaltrials.gov/ (NCT03132636, NCT03521830, and NCT04323202). One of the trials uses the recently approved cemiplimab (Libtayo^®^, Regeneron Pharmaceuticals Inc., Tarrytown, NY), a PD-1 inhibitor indicated to treat patients with laBCC or mBCC previously treated with an HHI or for whom an HHI is not appropriate [79]. The phase 2 data from this single-arm, open-label trial of cemiplimab demonstrated clinically meaningful and durable responses in patients with laBCC who have experienced PD on HHI therapy or were intolerant of prior HHI therapy. The ORR for patients with laBCC was 31% (*n* = 84; 95% CI, 21.0%–42.0%), with estimated duration of response exceeding 1 year in 85% of responders (95% CI, 60.5%–95.0%), and an estimated PFS of 19.0 months (95% CI, 44.3%–67.0%) [80]. Consequently, in patients with advanced BCC and resistance to HHI treatment, cemiplimab may provide these patients with an alternative treatment option.

Interestingly, in a study of three patients with laBCC who had complete response following treatment with vismodegib that was clinically and histologically confirmed, and then relapsed after discontinuing treatment, mutational analysis of the coding regions of sequenced genes in relapsed tumors was not significantly changed compared with tumor analyses prior to vismodegib treatment [81]. These results demonstrate that tumor relapse following vismodegib discontinuation retains the same mutational pattern as the baseline tumor, suggesting these tumors may be eligible for treatment rechallenge [81].

Overall HHI resistance, either intrinsic or acquired, continues to be a challenge in a subset of patients with advanced BCC, despite HHIs efficacy and tolerability in most patients. Mechanisms of resistance to HHIs include mutations in SMO, noncanonical cell identity switching resulting in resistant tumor cells, and non-canonical pathway crosstalk that may activate the Hedgehog pathway. Management of patients with HHI resistance can include switching HHI agents, combination therapy of HHIs and radiotherapy, photodynamic therapy, and targeting specific downstream effectors of the Hedgehog signaling pathway. Although these approaches to HHI resistance may be effective in some patients, further identification of biomarkers of resistance is needed. Identification of biomarkers of resistance or response would have the potential to improve the therapeutic efficacy of HHIs by personalizing treatment, and should be a future research goal for the treatment of advanced BCC.

## Abbreviations

AE: adverse event
BCC: basal cell carcinoma
BET: bromo and extra C-terminal
CI: confidence interval
DYRK1B: dual-specificity-tyrosine-phosphorylation-regulated kinase 1B
FDA: Food and Drug Administration
HDAC: histone deacetylases
HHI: Hedgehog pathway inhibitors
laBCC: locally advanced BCC
mBCC: metastatic BCC
MKL1: megakaryoblastic leukemia-1
ORR: objective response rate
PD: progressive disease
PD-1: programmed cell death protein 1
PDT: photodynamic therapy
PFS: progression-free survival
PI3K: phosphoinositide-3-kinase
pKi: affinity
PTCH1: patched homologue 1
SMO: Smoothened homologue
SRF: serum response factor
SUFU: suppressor of fused
TEAE: treatment-emergent AE
